# The great debate at “Immunotherapy Bridge 2018”, Naples, November 29th, 2018

**DOI:** 10.1186/s40425-019-0683-0

**Published:** 2019-08-15

**Authors:** Paolo A. Ascierto, Lisa H. Butterfield, Sandra Demaria, Robert L. Ferris, Gordon J. Freeman, Roger S. Lo, Alberto Mantovani, Paul Nathan, Omid Hamid, Katerina Politi, Igor Puzanov

**Affiliations:** 1 Unit of Melanoma, Cancer Immunotherapy and Innovative Therapy, Istituto Nazionale Tumori IRCCS Fondazione “G. Pascale”, Naples, Italy; 2 Parker Institute for Cancer Immunotherapy Research Center, UCSF, San Francisco, California USA; 3 Radiation Oncology, Department of Pathology, Weill Cornell Medical College, New York City, New York USA; 4 UPMC Hillman Cancer Center, Pittsburgh, PA USA; 5 Department of Medical Oncology, Dana-Farber Cancer Institute, Harvard Medical School, Boston, Massachussetts USA; 6 Melanoma Clinic, Jonsson Comprehensive Cancer Center David Geffen School of Medicine at UCLA, Los Angeles, California USA; 7 Humanitas University, Rozzano (Milan), Italy; 8 William Harvey Research Institute, Queen Mary University, London, UK; 9 Mount Vernon Cancer Centre, Northwood, Middlesex, UK; 10 Clinical Research and Immunotherapy, The Angeles Clinic and Research Institute, Los Angeles, California USA; 11 Department of Pathology and Yale Cancer Center, Yale University School of Medicine, New Haven, CT USA; 12 Department of Medicine, Roswell Park Comprehensive Cancer Center, Developmental Therapeutics, Buffalo, New York USA

**Keywords:** Anti-CTLA-4, Anti-PD-1, Cancer, Combination therapy, Immunotherapy, Immunity, Treatment resistance

## Abstract

As part of the 2018 Immunotherapy Bridge congress (November 28–29, Naples, Italy), the Great Debate session featured counterpoint views from leading experts on four topical clinical issues in immunotherapy today. These were: the relative importance of adaptive versus innate immunity in the anti-cancer immune response; the merits of combination versus sequential immunotherapy regimens in the treatment of cancer; the advantages and disadvantages of murine models of cancer versus humans in order to evaluate immunotherapy; and whether or not mechanisms of resistance to immunotherapy differ between different cancers. Discussion of these important topics are summarised in this report.

## Introduction

As part of the 2018 Immunotherapy Bridge congress (November 28–29, Naples, Italy), the Great Debate session featured counterpoint views from leading experts on four controversial clinical issues in immunotherapy today. The first topic was the relative importance of adaptive versus innate immunity in the anti-cancer immune response. While the immune response involves both innate and adaptive immune cells, immunotherapeutic strategies have primarily focused on stimulation of adaptive immunity. However, there is increasing recognition of the potential contribution of innate anti-tumor immunity, especially in the context of combination immunotherapy the second topic considered the merits of combination versus sequential immunotherapy in cancer. Immunotherapy has revolutionized the treatment of many advanced stage cancer but the increasing number of treatment options has increased the complexity of clinical decision-making. The respective benefits of combination immunotherapy versus sequential immunotherapy are not yet fully understood. Thirdly, the use of murine models versus humans to assess immunotherapy was discussed. Murine models can be used to provide information on many aspects of cancer immunotherapy, although to what extent these findings translate to humans is debatable and interrogating human specimens may provide more meaningful data. Finally, whether mechanisms of resistance to immunotherapy differ between different cancers was debated. Overcoming resistance to immunotherapy is a key consideration in improving outcomes for patients so better understanding of the mechanisms of resistance, including whether these ae the same or differ between cancers, is critical.

For each topic, two experts presented the argument and counter-argument in support of two different points of view. Note that these points of view may not have been entirely shared by the speaker; however, each speaker was asked to present a particular viewpoint. The views summarised in this article are based on available evidence but may reflect personal interpretation of these data, clinical experience and subjective opinion of the speaker. These perspectives are not intended to be a rigorous assessment of the topic and associated data but rather reflect two possible viewpoints and so provide the opportunity to consider different opinions. The audience were asked to vote on which view they supported both before and after the debate. Discussion of these important topics are summarised in this report.

## Which is more important: adaptive or innate immunity?

### Gordon J. freeman: in favour of adaptive immunity

T-cells are clearly responsible for killing tumor cells, while the innate immune system can be subverted to promote the growth of cancer via tumor recruitment of suppressive myeloid cells which actively promote cancer, something that T cells do not do. For example, breast cancer cells have been shown to recruit tumor-infiltrating myeloid cells via the cytokines interleukin (IL)-1α and t thymic stromal lymphopoietin (TSLP) to maintain their survival [1]. It is also clear that therapies targeted against T cells, in particular anti-PD-1/PD-L1 antibodies, are remarkably effective. The importance of T cells in the effectiveness of these treatments can be shown in mouse models. In an orthotopic, immunocompetent murine glioblastoma model, combination therapy of anti-CTLA-4 plus anti-PD-1 resulted in 75% survival, even with advanced, late-stage tumors [2]. Checkpoint blockade triggered a robust intra-tumoral T cell infiltration, which was especially seen with the anti-CTLA-4 plus anti-PD-1 combination. PD-1 blockade increased the number and functionality of intratumoral CD4 T cells. In vivo depletion experiments showed that both CD4 and CD8 T cells were required for response to PD-1 blockade. In contrast, depletion of natural killer (NK) cells did not reduce the efficacy of PD-1 blockade in this model. Enthusiasm for immunotherapy is in part based on T cells having a memory. In the murine model, tumor growth was not seen upon intracranial tumor rechallenge in long-term survivors, suggesting that tumor-specific immune memory responses were generated. This is reflected in the long-term duration of responses to ipilimumab and anti-PD-1 therapy seen in patients with melanoma. In contrast, targeted kinase inhibitors can achieve higher initial response rates but resistance usually develops. The anti-tumor adaptive immune response involves neoantigens being presented to T cell receptors. The importance of the adaptive immune response is confirmed by the finding that a high mutational load generally correlates with a higher rate of response to checkpoint blockade. Tumor mutational burden and a T cell-inflamed gene expression profile have shown joint predictive utility in identifying responders to anti-PD-1 blockade, with both independently predictive of response [3]. B cells also have a role in the adaptive immune response. B-cells and the development of tertiary lymphoid structures within the tumor predict response to immune checkpoint blockade [4]. Intratumoral B cells are present as activated, class switched effector cells and may be contributing to response by either antibody production or antigen presentation to facilitate T cell function.

Current immunotherapies are effective in patients with a pre-existing anti-tumor immune response and the challenge is to bring immune cells into tumors that are an immune desert. The future is clearly PD-1/PD-L1-based combination approaches, including with other checkpoint inhibitors (e.g. CTLA-4, T-cell immunoglobulin and mucin-domain containing [TIM]-3, lymphocyte-activating gene [LAG]-3, T cell immunoreceptor with Ig and ITIM domains [TIGIT]), immunostimulators (e.g. OX40, CD137, IL-15, toll-like receptor [TLR] ligands, STING), and myeloid targets (e.g. CD47, CSFR1, Indoleamine-pyrrole 2,3-dioxygenase [IDO], arginase, chemokines). The latter myeloid targets briefly mentioned here impact on adaptive responses as discussed below by Alberto Mantovani.

### Alberto Mantovani: in favour of innate immunity

Inflammation is a manifestation of innate immunity and a key component of the tumor microenvironment (TME). Factors linking inflammation and cancer can be at a tissue level (e.g. carcinogen oncogene activation, chronic non-resolving inflammation) and a systemic level (e.g. obesity, aging) with both leading to inflammation and tumor promotion. Macrophages are key drivers of tumor-promoting inflammation and represent a final common pathway driving cancer-related inflammation. Tumor-associated macrophages (TAMs) contribute to tumor progression at different levels, including stimulating tumor cell proliferation, migration and genetic instability, promoting invasion and metastasis, and suppressing adaptive immunity by the expression of immunosuppressive molecules, such as IDO, cyclooxygenases (COX1,2), transforming growth factor [TGF]-β and IL-10 [5].

Moreover, TAMs can contribute to creating an immunosuppressive environment in tumors through multiple routes, including triggers of checkpoint blockade, and thus represent targets of checkpoint blockade immunotherapy. Macrophages express the ligands for checkpoint molecules, including PD-L1, PD-L2, and the CTLA-4 ligands B7–1 and B7–2. PD-L1 and PD-L2 are upregulated in response to various stimuli including cytokines and hypoxia. It has not been fully elucidated how and to what extent the expression of inhibitory receptors on macrophages contributes to their immunosuppressive function.

IL-1 is an inflammatory cytokine which plays a key role in carcinogenesis and tumor progression, including driving chronic non-resolving inflammation, tumor angiogenesis, activation of the IL-17 pathway, induction of myeloid-derived suppressor cells (MDSCs) and macrophage recruitment, invasion and metastasis. Initial evidence suggests that targeting the innate immunity pathway through IL-1β inhibition with canakinumab could significantly reduce incident lung cancer and lung cancer mortality in patients treated for atherosclerosis [6].

Innate myeloid cells also interact with NK cells, innate lymphoid cells which contribute to the activation and orientation of adaptive immune responses. NK cells engage in a complex bidirectional interaction with myelomonocytic cells. In particular, macrophages, dendritic cells and neutrophils promote differentiation and effector function of NK cells and, on the other hand, myelomonocytic cells express triggers of checkpoint blockade (e.g. PD-L1) and other immunosuppressive molecules, which negatively regulate NK cell function. In addition, NK cells express high levels of IL-1 receptor 8, which serves as a checkpoint for NK cell maturation and effector function, and its blockade unleashes NK-cell-mediated resistance against solid tumors at NK-rich anatomical sites [7].

T cell-centred immunotherapy has clearly been revolutionary in cancer treatment, but targeting myeloid cells is important and it is possible that immunotherapies targeted against innate immunity represent a major strategy in the future. Promising results have been obtained recently targeting the macrophage CD47-SIRP checkpoint axis in non-Hodgkin lymphoma in combination with rituximab [8, 9].

Innate immunity and inflammation thus play a fundamental role in tumor progression and as therapeutic targets. Moreover, one should emphasize that suppression of tumor promoting inflammation or unleashing the antitumor potential of macrophages will eventually impact the activation and expansion of adaptive immune responses.

## Key points

**Fig. 1:**
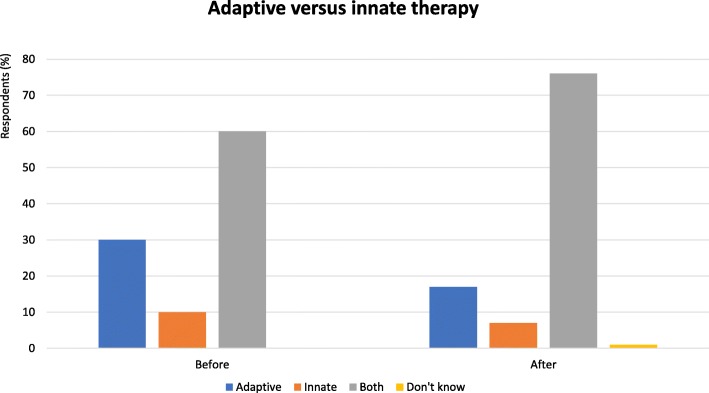
Adaptive versus innate immunity - Proportion of audience who considered adaptive or innate immunity as more important in the immune response. Audience response before and after debate

- The adaptive immunity landscape, T cells in particular, of tumors is an important prognostic indicator.
- T cell infiltration is associated to response to checkpoint blockade therapy.
- New checkpoints and their combinations hold promise.
- Inflammation is a component of the tumor microenvironment.
- Macrophages, other inflammatory cells and inflammatory cytokines, IL-1 in particular, promote progression and metastasis.
- Checkpoints of innate lymphoid cells and macrophages hold promise to provide new therapeutic strategies.
- Unleashing myeloid cells or blocking their suppressive function has the potential to complement T cell centered immunotherapies (Fig. 1).

## What treatment approach is more useful: combination therapy versus sequential therapy?

### Paul Nathan: in favour of combination therapy

Historically, combinations have been widely used in cancer. Combination chemotherapy regimens are more likely to delay tumor escape and offer an improved palliative benefit while an improved curative benefit has been seen in an adjuvant setting so there is a clear precedent for combination therapy. With regard to immunotherapy, the two main combination approaches are combining different immune checkpoint inhibitors and combining a checkpoint inhibitor with targeted therapy, where the goal is to increase the proportion of patients who gain durable benefit from their immunotherapy through a change in the TME induced by targeted agents.

In the CheckMate 067 study, 4-year overall survival (OS) rate was 53% with nivolumab plus ipilimumab, 46% with nivolumab alone and 30% with ipilimumab alone [10]. The incremental benefit of combination nivolumab plus ipilimumab versus nivolumab in terms of PFS is approximately the same order of magnitude as single agent ipilimumab. There is therefore no evidence of a synergistic effect and therefor one could argue there is possibly no benefit over sequential single agent therapy.

However, response rates are higher with combination therapy and there appears to be an association between response rate and the proportion of patients who have durable benefit with immunotherapy. In addition, disease progression is associated with an increase in lactate dehydrogenase (LDH), an increased number of organ sites, more involvement of critical organs and a decrease in performance status. All of these prognostic biomarkers worsen with time and therefore it is possible that the likelihood of benefit from a second line agent would not be as great as if it were given in combination first line due to a deterioration of prognostic biomarkers. The risk of side effects from single agent first line therapy can also that mean second-line treatment is compromised. Combination first line treatment has higher toxicity rates however the patient and their disease are exposed to both agents even if toxicity in dose limiting. A minority of patients will have such significant toxicity from first line single agent drug exposure that it limits the possibility of second line treatment. Thus, sequencing means fewer patients will have the opportunity to receive both drugs.

Pre-clinical data show that combining anti-PD-1 therapy with targeted therapy (dabrafenib plus trametinib) provides superior anti-tumor activity versus anti-PD-1 plus either therapy alone. In patients, there is evidence of immune activation after treatment initiation with the anti-PD-1 antibody spartalizumab in combination with dabrafenib and trametinib in patients with advanced BRAF-mutant melanoma with a significant increase in intratumoral CD8+ cells and elevated interferon (IFN)-γ levels in plasma upon treatment [11]. In the double-blind KEYNOTE-022, patients with treatment BRAF-mutant melanoma were randomized to triple combination therapy of pembrolizumab plus dabrafenib plus trametinib or placebo plus dabrafenib plus trametinib [12]. Median progression-free survival (PFS) was 16.0 months (95% CI 8.6–21.5) with the triple combination versus 10.3 months (95% CI 7.0–15.6) with dabrafenib plus trametinib (hazard ratio [HR] 0.66). This was not statistically significant according to the study design and more follow-up is required to determine whether the pembrolizumab plateau is elevated by exposure to dabrafenib and trametinib.

Comparing across studies, combination treatments appear to be more beneficial. In a survival analysis of metastatic melanoma clinical trials, combined PD-1 plus CTLA-4 inhibition demonstrated the best survival outcome in all categories except for OS in first-line therapy [13]. However, there are risks involved in combining treatments. There can be a tendency to inherit combinations that have not been proven to be superior to sequencing. Typically, two drugs are usually better than one and so can become a standard of care without sufficient supportive evidence. This can mean ethical issues in performing future studies without using the combination. However, despite these concerns, combinations look to be the way forward. Novel combination therapies are more likely than sequential therapy to significantly increase the proportion of patients having durable benefit. There is a need to evaluate the biological hypothesis in early clinical studies and conduct combination studies where there is a strong pre-clinical rationale. It is also important to ensure tolerability of the tested combination.

### Robert L. Ferris: in favour of sequencing therapy

Although evidence suggests that combination regimens are better than monotherapy, this is only true if we know which patients should receive which combination. While not all patients benefit from monotherapy, some can benefit substantially and treating using a combination regimen may increase toxicity without any additional clinical benefit. It should also be noted that combinations are not restricted to immunotherapies but also include how immunotherapy is best integrated with chemotherapy and radiotherapy. Moreover, the financial cost of combinations can be prohibitive and means they are impractical for treating all patients.

Combination therapy may not always be better than monotherapy. In the CheckMate-067 trial, median PFS was 11.5 months with nivolumab plus ipilimumab as compared with 2.9 months with ipilimumab (HR for death or disease progression, 0.42; *P* < 0.001), and 6.9 months with nivolumab (HR for the comparison with ipilimumab, 0.57; P < 0.001) [14]. However, the benefit of combined nivolumab plus ipilimumab only applied to the subgroup with PD-L1-negative tumors. In these patients, PFS was longer with the combination therapy than with nivolumab alone (11.2 vs. 5.3 months). In patients with positive PD-L1 expression, however, there was no difference in median PFS between the combination and with nivolumab alone (both 14.0 months) so there was no real benefit of adding a second therapy. In addition, grade 3–4 treatment-related adverse events occurred in 55% of patients in the combination group, more than in the two monotherapy groups combined (nivolumab, 16%; ipilimumab 27%). Thus, the increased toxicity of the combination was more than multiplicative, whereas the oncological benefit was not even additive. From a patient perspective, this may mean that the small clinical benefit from the combination does not outweigh the increased side effects. This consideration, together with the increased cost, suggests combination therapy may not always be the appropriate choice.

Sequencing can also allow increased understanding of the biology of response and how immunotherapies work with other treatments. In the CheckMate-141 trial of patients with recurrent or metastatic squamous-cell carcinoma of the head and neck (SCCHN), prior exposure to cetuximab dramatically reduced the benefit of subsequent nivolumab therapy [15]. If patients had been given both treatments in combination, this may not have been so apparent. The PACIFIC trial has shown that positive survival outcomes can be achieved with anti-PD-L1 therapy after chemoradiation in patients with non-small-cell lung cancer (NSCLC) [16]. Combined checkpoint inhibition and chemoradiation may have resulted in more potential toxicity. Recent data have also suggested that checkpoint inhibition treatment can potentially improve response to salvage chemotherapy. In a study of patients with SCCHN treated with salvage chemotherapy after progression on immune checkpoint inhibitor therapy, a response rate of 30% was observed, suggesting that immunotherapy may increase tumor sensitivity and unlock therapeutic benefit from conventional chemotherapy [17]. Similar results have been seen in NSCLC [18]. Similar results have been seen in NSCLC (Schvartsman, Lung Cancer October (2017) 112: 90–95.

In the KEYNOTE-048 study in patients with SCCHN, the response rate with pembrolizumab plus chemotherapy was similar to that of chemotherapy without pembrolizumab [19]. Thus, pembrolizumab might be better used in a sequential manner by being given to patients who have progressed on chemotherapy, since using in combination may effectively forego any additional benefit of immunotherapy. Another example is provided by a retrospective series of BRAF-mutated patients treated either with BRAF inhibitor first or ipilimumab first [20]. Improved OS was observed in patients treated with immunotherapy first (14.5 vs. 9.9 months, *p* = 0.04). After BRAF inhibitor, 40% were rapid progressors and were unable to complete four courses of ipilimumab. However, caution should be taken when interpreting these results as patients without brain metastasis and normal LDH were selected to receive immunotherapy first.

In conclusion, monotherapy benefits many patients and allows the identification of biomarkers and greater understanding of biological processes. Combinations may be additive or multiplicative in toxicity but not in oncological benefit. Most importantly, the cost of combination therapies is such that their use is unaffordable for all patients that might benefit. However, more data from well designed robust combination and sequencing studies are required to definitively address this question.

## Key points

**Fig. 2:**
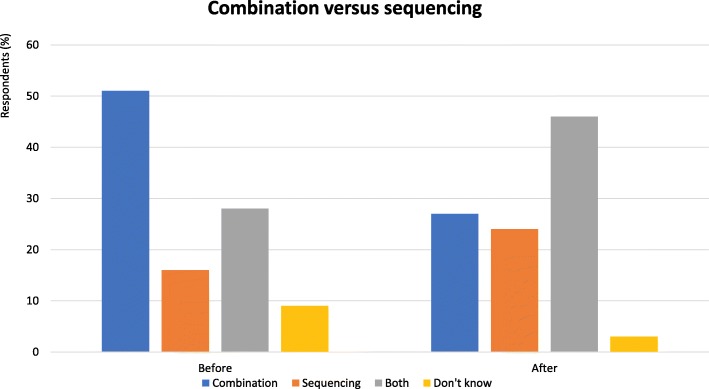
Combination versus sequencing - Proportion of audience who considered combination or sequential therapy more useful. Audience response before and after debate

- Combination immunotherapy can add therapeutic benefit but usually substantially increases toxicity. However, when treating with the intent of gaining long term durable disease control, the additional activity of combination immunotherapy may justify additional acute toxicity for many patients.
- First line combination immunotherapy avoids a reduction in the chance of benefiting from 2nd line treatment due to deteriorating prognosis.
- The health economics of first line combination treatment are not inferior to sequential treatment due to the fact most patients have extended treatment duration with single agent therapy.
- Subsets of patients who derive benefit from combination immunotherapy are not well defined, increasing toxicity and cost if one treats all-comers
- Sequencing immunotherapeutic permits more detailed investigations into resistance mechanisms and rational combinations (Fig. 2).

## Which is the most useful preclinical model: mouse or human?

### Roger Lo: in favour of mouse models

It is clear that the large number of immunotherapy-based combinations shows a lack of prioritization based on scientific merits. It is also not possible to study all possible combinations in enough patients quickly. Mouse models can offer certain advantages that cannot readily be obtained from studying patients or patient samples. These include the provision of data that can help to rationally discern which combinations are likely to be the most clinically useful, as well as the ability to elucidate mechanistic processes, identify biomarkers to enrich patient cohorts for treatment, and the possibility to differentiate between simple correlations and causality in tumor processes.

Efficacy of and resistance to targeted therapy with BRAF and MEK inhibitors are influenced by anti-tumor immunity [21–23]. Mitogen-activated protein kinase (MAPK) inhibitors induce immune-suppressive pathways, which may exclude or exhaust tumor antigen-specific CD8 T-cells that infiltrate the MAPKi-treated tumors. Differences between innate PD-1 resistance and sensitive melanoma samples show the importance of tumor mutational burden. Certain transcriptional signatures are associated with innate PD-1 resistance. These signatures relate to a number of biological processes that have been recapitulated in mouse models. Thus, while targeted therapy can induce T cells and has the potential to improve response to checkpoint inhibitors, concomitant signatures and processes induced by targeted therapy can also be antagonistic to immunotherapy efficacy.

The potential of triple combined therapy with a BRAF inhibitor, MEK inhibitor and anti-PD-1 agent has been studied in mice to help elucidate whether the combination is truly synergistic or additive. Different BRAF, NRAS, Nf1 and KRAS mutant-driven syngeneic mouse melanoma models have been created and characterized. In murine syngeneic mutant-Braf melanoma mice without high mutational load, BRAF inhibitor exposure resulted in residual tumors followed by acquisition of resistance as shown by tumor growth [23]. Loss of T cells was observed after the development of acquired resistance. Increase in innate anti-PD-1 resistance (IPRES) signature preceded loss of T cell inflammation, suggestive of causality. In murine melanoma with a high mutational burden, complete responses and anti-melanoma immunologic memory is possible. CD8 T-cells suppress resistance development to MAPK inhibitors in BRAF and NRAS-mutant melanoma with high mutational burden and targeting IPRES can enhance the anti-tumor activity of combined BRAF inhibitor and anti-PD-1 antibody. The use of mouse models can help elucidate mechanistic processes and the sources of signatures and offers the ability to assess at multiple timepoints along the course of a complex, evolutionary process subject, which would be difficult and take several years in humans. Mouse models can also help understand how individual components of tumors contribute to resistance and allow better understanding of causality based on the sequence of observed events. In conclusion, clinically relevant mouse models can help to understand mechanistic processes, including the difference between causality and simple correlation and provide in vivo proof-of-concept for different combination therapy approaches.

#### Omid Hamid: in favour of human models

In melanoma, approximately 80% durable complete responses have been obtained with the triple combination of the histone deacetylase (HDAC) inhibitor domatinostat plus anti-PD-1/LAG3 blockade in mouse models. Similarly, the IDO inhibitor epacadostat suppressed tumor growth in immunocompetent mice. However, in clinical studies, pembrolizumab plus epacadostat was no more effective than pembrolizumab alone, indicating that although IDO inhibition worked in mice it was not effective in patients. In general, mouse models to date have not identified biomarkers or offered a good path forward.

T-cell tolerance mechanisms, and immune-escape routes of tumors come from in vivo studies with cell line allograft models. These findings have laid the foundation for the currently ongoing cancer immunotherapy revolution. However, there are patients across multiple cancers who have not shown robust responses to these agents. A major impediment to progress in the field is the lack of mouse models that reflect the complexity of human malignancy and immune contexture within the TME. The way forward is to interrogate and predict antitumor immune responses and therapeutic efficacy in clinical trials and then create mouse models to evaluate what is initially found in humans.

Syngeneic tumor lines are fully immunocompetent and useful in the evaluation of immunoncology agents and to study the generation of de novo antitumor immune responses. However, there are issues regarding tumor penetrance and latency, as well as the lack of shared tumor antigens. These models can be used for studies that require large group numbers that are difficult to obtain using genetically engineered models or patient-derived xenografts. However, they lack genomic and microenvironmental heterogeneity and mutational patterns that recapitulate human intra-patient genomic heterogeneity. Moreover, they are implanted into a limited number of inbred strains of mice that lack the interpatient heterogeneity (few transplantable cell lines) and there is a lack of native TME. There is also the variability of phenotype depending on the site of engraftment as well as lower levels of genomic instability. As such, they are poor in helping us understand the complexity of initial tumor growth and they do not undergo the natural steps of tumor progression (i.e. premalignant transformation, tumor development, and progression) as occurs in humans. They also do not permit the evaluation of immunotherapy in earlier stages of disease, which may potentially be the optimal time point to initiate immunotherapeutic intervention.

Genetically engineered mouse models with incorporation of specific genomic alterations to provide autochthonous tumor development in a tissue-specific manner are important but only work for evaluating oncogenes. They have successfully been used to validate candidate cancer genes and drug targets and to assess therapy efficacy. However, overexpression or deletion of a select number of genes and the tumor mutational burden may not replicate that seen in humans, with less neoepitopes and targeting of specific genes to promote tumorigenesis/accumulation mutations.

Instead of these models, we should be focused on humanized tumor models based on data collected in clinical trials. Patient-derived xenografts can more closely reproduce the complexity of human disease (genomic heterogeneity, cell types) and do not require immune reconstitution. However, disadvantages include that evaluation is conducted in an immune-deficient host, they rely on human immune cells being transferred, the murine stroma as well as a low implantation rate and high cost. Long-term engraftment may provide an answer. Hematopoietic progenitor-rich populations are modified to incorporate chemokines and other agents and stimulate the generation of stromal cells and the formation of the TME and tumor-infiltrating lymphocytes (TILs). Patient-derived xenograft-bearing mice have been shown to recapitulate antitumor responses seen in patients but are limited to malignancies in which sufficient amounts of TILs can be made.

Genomic responses in mice poorly mimic human inflammatory disease and treatment responses in mice are not necessarily reflected in humans. A better approach would be parallel studies in humans and mouse models. Initial studies in mice to validate cancer drivers and drug targets should be combined with phase I/II trials and in vivo testing of drug efficacy to assess resistance and treatment failures and then evaluation of combination therapies in enriched patient cohorts. This approach has been shown in microbiome studies, where the optimal microbiome identified in humans was implanted into mice for further investigation.

In conclusion, mouse models can provide preliminary data on efficacy, toxicity and pharmacokinetics but interrogating human specimens is necessary to move the field forward. Human specimens derived from studies need to be interrogated and then mouse models used to help evaluate responses in the tumor and the antitumor response in the immune system and help identify appropriate biomarkers.

## Key points

**Fig. 3:**
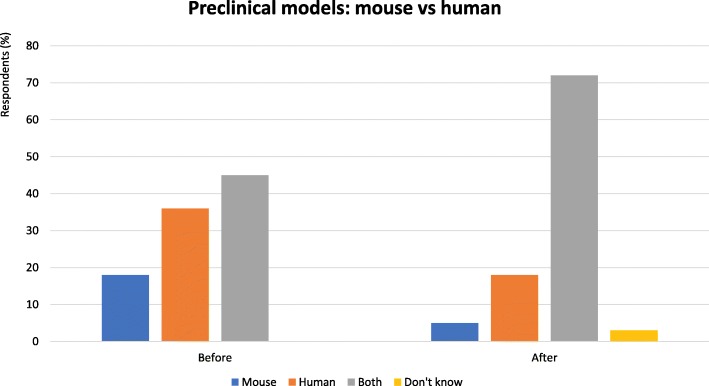
Preclinical models: mouse vs human - Proportion of audience who considered mouse or human preclinical models the most useful. Audience response before and after debate

- Mouse models can provide data to help discern which combinations are likely to be the most clinically useful, elucidate mechanistic processes, identify biomarkers to enrich patient cohorts for treatment, and also offer the possibility to differentiate between simple correlations and causality in tumor processes.
- Mouse models to date have not identified biomarkers or offered a good path forward.
- A major impediment to progress in the field is the lack of mouse models that reflect the complexity of human malignancy and immune contexture within the TME.
- Patient-derived xenografts can more closely reproduce the complexity of human disease (genomic heterogeneity, cell types) and do not require immune reconstitution.
- Mouse models can provide preliminary data on efficacy, toxicity and pharmacokinetics but interrogating human specimens is necessary to move the field forward (Fig. 3).

## Is resistance different in different cancers? Yes or no

### Sandra Demaria: yes, resistance is different in different cancers

Resistance to immunotherapy may be primary, adaptive or acquired. The cancer immunoediting model [24] offers a framework by which to understand interactions between tumor and the immune system but it is clear that not all tumors interact in the same way with the immune system. Tumors need to escape control by the immune system in order to grow and disseminate, and this escape can be achieved in many different ways resulting in different immunophenotypes. When tumors become clinically apparent the more immunogenic cells have been edited out and the cells that are left survive by decreasing antigen expression or by inhibiting T cells.

Three major tumor immunophenotypes have been defined. Infiltrated-inflamed tumors are characterized by high infiltration of cytotoxic lymphocytes expressing PD-1 and leukocytes and tumor cells expressing PD-L1. A subset of infiltrated-inflamed tumors displays evidence of tertiary lymphoid structures (TLSs), lymphoid aggregates with a cellular composition similar to that in lymph nodes, that are often correlated with a better prognosis [25]. This immunophenotype is the most responsive to immune checkpoint inhibition. Tumors that are broadly populated with immune cells but in which T cells are present at the periphery of the tumor and do not penetrate into cancer cell areas have been termed immune-excluded. The third phenotype, has been defined as immune desert because it shows little evidence of immune infiltration.

Importantly, the same Immunophenotype may result from different mechanisms. For example, the excluded phenotype in urothelial cancer was shown to be dependent on TGF-β signaling in tumor-associated fibroblasts [26]. In contrast, in a pancreatic cancer model, tumor cell-derived CXCL1 precluded T cell infiltration. Moreover, identical tumor-initiating alterations in pancreatic cancer were shown to give rise to different dominant mechanisms of immune exclusion [27]. Thus, mechanisms of immune exclusion are themselves heterogeneous, even within a genetically homogeneous cancer model.

Oncogene activation has been linked to aberrant production of cytokines and chemokines that shape the tumor immunophenotype. For example, BRAFV600E mutation in a PTEN deficient melanoma induced constitutive Wnt/β-catenin signalling, which in turn decreased production of CCL4, precluding dendritic cell (DC) and T cell recruitment to the tumor [28, 29]. In KRASG12D-driven pancreatic adenocarcinoma high levels of granulocyte-macrophage colony-stimulating factor (GM-CSF) led to recruitment of immunosuppressive myeloid cells [30, 31].

Turan et al. [32] have analysed various gene signatures, such as the Immunological Constant of Rejection (ICR), in an attempt to delineate the nature of the different TMEs. ICR groups are ranked from 1 to 4 based on the level of expression of the 20 representative ICR genes and the distribution of Signatures of Responsiveness (sRes) according to distinct models. Clustering of transcriptional sRes demonstrated a preferential distribution of immune suppressive functions in the ICR3 and ICR4 groups (immune-active), whereas ICR1 and ICR2 were immune-depleted (immune-silent). Overall, they suggest a dichotomy of mechanisms of tumor immune escape: Immune-active tumors, are highly genetically unstable, generate a lot of mutations and stress-related danger signals, and become inevitably visible to the immune system as an aberrant tissue. These tumors resist immune rejection via multiple immunosuppressive mechanisms. In contrast, immune-silent (cold) tumors are more likely to be oncogene-addicted and avoid generating danger signals that activate the innate immune system. For the latter, therapeutic interventions such as radiation that cause DNA damage, cell stress and the release of danger signals may be required to jump-start immune recognition.

In conclusion, there are many mechanisms of resistance, that can be considered tumor cell intrinsic or extrinsic [33]. Intrinsic mechanisms include the absence of antigenic proteins (e.g. low mutational burden, lack of viral antigens), absence of antigen presentation (e.g. deletion in TAP, beta-2-microglobulin [B2M], silenced human leukocyte antigen [HLA]) or genetic T cell exclusion (e.g. MAPK oncogenic signalling stabilized β-catenin mesenchymal transcriptome oncogenic PD-L1 expression) or insensivity to T cells (e.g. caused by mutations in interferon gamma pathway signalling). Extrinsic mechanisms include absence of T cells (e.g. lack of T cells with a T cell receptor in the repertoire that can recognize the expressed tumor antigens), inhibitory immune checkpoints (e.g. V-domain Ig suppressor of T cell activation [VISTA], LAG-3, TIM-3) or the presence of immunosuppressive cells (e.g. TAMs, T regulatory cells [Tregs]). Finally, many host and environmental factors modulate tumor immune resistance. The concept of a patient-specific cancer immune setpoint takes into consideration the baseline characteristics of a given tumour in the context of host germline genetics, age, microbiome, and other factors that can influence the ability of the immune system to fight the tumor, including infectious agents, exposure to sunlight and pharmacological agents [34].

### Katerina Politi: no, resistance is the same in different cancers

Primary and acquired resistance to immunotherapies is a major clinical problem. Response rates are very variable across different tumor types and many tumors do not respond to immunotherapy highlighting the problem of primary resistance [35]. Acquired resistance to immune checkpoint inhibitors is also a frequent challenge despite durable responses in many patients. Although the exact frequencies remain to be determined, acquired resistance is estimated to occur in around 30% of patients with melanoma and 50–70% of patients with lung cancer and microsatellite instability-high (MSI-H) colorectal cancer (see for example, Herbst et al. [36]).

Two common tumour cell-intrinsic determinants of sensitivity and resistance to checkpoint blockade are tumor cell recognition by the immune system and tumor-mediated immune suppression and exclusion. Tumor mutation burden is an important component in determining response to checkpoint inhibitors across different cancers [37]. This is illustrated by the recent approval of pembrolizumab for patients with MSI-H or mismatch repair deficient (dMMR) solid tumors, which is the first US Food and Drug Administration (FDA) approval based on a biomarker rather than the type of cancer. This approval is consistent with the concept that tumors with higher mutational burden are more likely to be responsive to immunotherapy.

Similar patterns of response to immune checkpoint blockade have been observed in models with elevated tumor mutational burden. In YUMM melanoma cell line-derived tumors which have a low mutational burden, immune checkpoint inhibitors are ineffective. However, a response is seen in tumors derived from the UV-irradiated YUMMER cell line mice which has a higher mutational burden [38]. Another common determinant of sensitivity/resistance across different tumor types is HLA presentation, an important mechanism of tumor cell recognition by the immune system. Defects in all steps in the processing and presentation of major histocompatibility complex (MHC) class 1 antigen are associated with resistance to immune checkpoint inhibitors. For example, loss of function mutations in and genomic loss of *B2M*, an essential component of MHC class I antigen presentation machinery, have been linked with resistance across several tumors, including colorectal cancer, melanoma and lung cancer [39–42].

In addition to genomic alterations, downregulation of HLA 1 antigen presentation can also result in resistance. Transcriptional suppression of specific HLA genes was associated with resistance to immune checkpoint therapy and relapse in two patients with metastatic Merkel cell carcinoma [43]. Similarly, downregulation of B2M was found in lung cancer patient-derived xenografts from immune checkpoint inhibitor-resistant tumors [40].

Although these data suggest that HLA class I antigen presentation disruption can mediate escape from immune checkpoint inhibitors across cancer types, the functional significance of many alterations in MHC 1 genes remains to be determined. Resistance may be irreversible (e.g. due to B2M/HLA gene mutation or neoantigen loss) or reversible (e.g. due to immune inhibitory signalling or epigenetic silencing of MHC I genes) which has implications for how tumors are treated and how resistance is overcome. If irreversible, MHC 1 independent therapies may be required to overcome resistance which could include leveraging innate immune cells like NK cells or myeloid cells or using engineered T cells. However, downregulation of the antigen presentation machinery may be reversible and treatments to reinvigorate T cells (e.g. cytokines, epigenetic drugs) may be an option.

Another common mechanism of resistance is tumor-mediated immune suppression or exclusion. An example of this is derived from oncogenic pathways in tumors that promote resistance to the antitumor immune responses. Alterations in PTEN are associated with immunotherapy resistance across tumor types. In melanoma models and patients, PTEN loss is associated with increases in immunosuppressive cytokines, decreased T-cell infiltration at tumor sites and worse outcomes with anti-PD-1 inhibitor treatment [44]. In metastatic uterine leiomyosarcoma, loss of PTEN was associated with resistance to anti-PD-1 inhibitor therapy [45].

To conclude, there are clearly shared mechanisms of resistance across different tumors. Understanding the type of mechanism that leads to resistance may be important in selecting approaches to overcome resistance. However, differences in mechanisms between and within cancers also exist.

## Key points

**Fig. 4:**
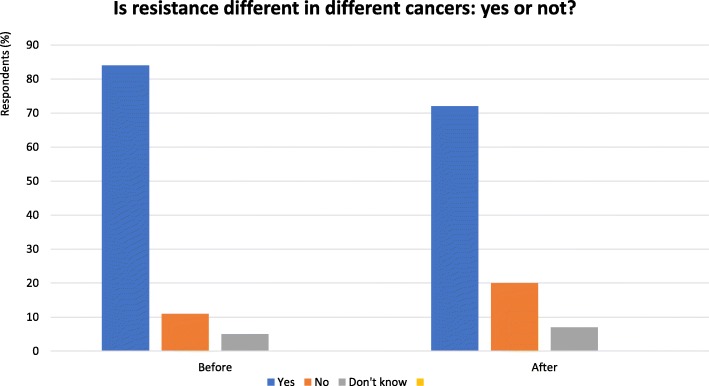
Is resistance different in different cancers? Yes or no - Audience response before and after debate. Audience response before and after debate

- Cancer resistance to immunotherapy can be primary or acquired during treatment.
- Cancers that become clinically apparent have all escaped immune control but in different ways, resulting in three major tumor immunophenotypes.
- The mechanisms resulting in each of these major tumor immunophenotypes can be different in different tumors.
- Common determinants of resistance across cancers include the ability of immune cells to recognize tumor cells and tumor mediated mechanisms of immune suppression or exclusion.
- Tumor cell recognition by immune cells depends on the tumor mutation burden and on the ability of the tumor cells to present antigens. The status of both of these can influence sensitivity to T-cell directed therapies across several cancers.
- Tumor intrinsic alterations in oncogenic pathways (e.g. PTEN) can affect the tumor immune microenvironment by altering cytokine levels and immune cell infiltration and thus contribute to resistance (Fig. 4).

## Conclusions

Counterpoint views from leading experts on four controversial clinical issues in immunotherapy today were presented during these Great Debate sessions. Given the constraints of the format and the intended nature of the session, each presentation was not intended as a rigorous assessment of the field but rather provided an opportunity to highlight some important areas of debate within immunotherapy. It may be that there are no clear right or wrong answers to these questions; however, it is hoped that these discussions can help focus attention on these issues, stimulating further debate and encouraging the research needed to improve our understanding of immunotherapy and thereby further improve outcomes for patients.

## Data Availability

Not applicable.

## Abbreviations

B2M: beta-2-microglobulin
COX: Cyclooxygenase
CTLA-4: Cytotoxic T-lymphocyte-associated antigen
DC: Dendritic cell
dMMR: Mismatch repair deficient
FDA: Food and Drug Administration
GM-CSF: Granulocyte-macrophage colony-stimulating factor
HDAC: Histone deacetylase
HLA: Human leukocyte antigen
HR: Hazard ratio
ICR: Immunological Constant of Rejection
IDO: Indoleamine-pyrrole 2,3-dioxygenase
IFN: Interferon
IL: Interleukin
IPRES: Innate anti-PD-1 resistance
LAG-3: Lymphocyte-activating gene-3
LDH: Lactate dehydrogenase
MAPK: Mitogen-activated protein kinases
MDSC: Myeloid-derived suppressor cells
MHC: Major histocompatibility complex
MSI-H: Microsatellite instability-high
NK: Natural killer
NSCLC: Non-small-cell lung cancer
OS: Overall survival
PD-1: programmed death-1
PD-L1: Programmed death ligand-1
PFS: Progression-free survival
SCCHN: Squamous-cell carcinoma of the head and neck
sRes: Signatures of Responsiveness
TAMs: Tumor-associated macrophages
TGF: Transforming growth factor
TIGIT: T cell immunoreceptor with Ig and ITIM domains
TILs: Tumor-infiltrating lymphocytes
TIM-3: T-cell immunoglobulin and mucin-domain containing-3
TLR: Toll-like receptor
TLSs: Tertiary lymphoid structures
TME: Tumor microenvironment
Treg: T regulatory cell
TSLP: Thymic stromal lymphopoietin
VISTA: V-domain Ig suppressor of T cell activation
